# Retraction: Li, X.; et al. Circular RNA circ-FoxO3 Inhibits Myoblast Cells Differentiation. *Cells* 2019, *8*, 616

**DOI:** 10.3390/cells9112504

**Published:** 2020-11-19

**Authors:** Cells Editorial Office

**Affiliations:** MDPI, St. Alban-Anlage 66, 4052 Basel, Switzerland; cells@mdpi.com

It has come to our attention that two images in Figure 2A (100%), and Figure 2C (GM) of the manuscript [1] appear to be identical. The original figure is shown below:

**Figure 2:**
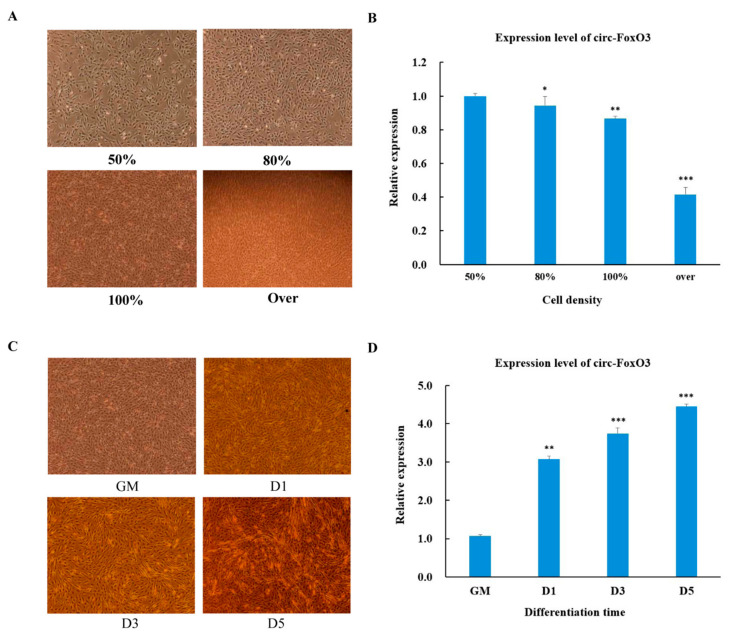
Expression pattern of circ-FoxO3 during C2C12 myoblast cells’ proliferation and differentiation. (**A**) Cell morphology of C2C12 myoblast cells at different densities (50%, 80%, 100%, and over). (**B**) Detection of circ-FoxO3 expression levels in C2C12 myoblast cells of different densities (50%, 80%, 100%, and over) by RT-qPCR. We used the β-actin gene as an internal reference to normalize the relative expression levels of circ-foxO3 at different cell densities, and we used circ-foxO3 expression levels in samples with a cell density of 50% (set to 1) to quantify other samples. (**C**) Cell morphology of C2C12 myoblast cells at different differentiation times (GM, D1, D3, and D5). (**D**) Detection of circ-FoxO3 expression in C2C12 myoblast cells at different time points by RT-qPCR (GM, D1, D3, and D5). GM represents an undifferentiated state. D1, D3, and D5 represent the first day of differentiation, the third day of differentiation, and the fifth day of differentiation, respectively. We used the β-actin gene as an internal reference to normalize the relative expression levels of circ-foxO3 at different differentiation times, and we used circ-foxO3 expression levels in GM samples (set to 1) to quantify other samples. All groups were performed with three biological replicates and all reactions were performed in triplicate. Error bars indicate ± SD, * *p* < 0.05, ** *p* < 0.01, and *** *p* < 0.001.

The authors provided explanations for the similarities that were not accepted by the journal editor. After thorough investigation, the journal has made the decision to retract the paper [1]. The retraction of the article was approved by the Academic Editor of *Cells*.

MDPI is a member of the Committee on Publication Ethics and takes its responsibility to publish only high-quality research seriously. We regret that these issues were not identified earlier and apologize to the readers of the journal.
